# Lightweight traffic sign detection algorithm with noise suppression and semantic enhancement

**DOI:** 10.1371/journal.pone.0340810

**Published:** 2026-01-21

**Authors:** Lingchao Wang, Qiang Ai, Haibo Jin

**Affiliations:** 1 College of Information Engineering, Liaoning Institute of Science and Engineering, JinZhou, China; 2 College of Computer, Qinghai Normal University, Xining, China; 3 School of Information Science and Engineering, Lanzhou University, Lanzhou, China; 4 School of Software, Liaoning Technical University, Huludao, China; Kafkas University: Kafkas Universitesi, TÜRKIYE

## Abstract

Existing traffic sign detection algorithms, when deployed on edge computing devices, often face challenges such as high computational overhead and insufficient detection capabilities for small or deformed targets. To address these limitations, this paper proposes LNSE-YOLO, a lightweight traffic sign detection algorithm based on YOLOv11, which incorporates novel noise suppression and semantic enhancement techniques. Initially, building upon the YOLOv11n architecture, we design a Feature Fusion Module named NSSE (Noise Suppression and Semantic Enhancement) to optimize multi-scale feature representation and effectively mitigate background interference. Subsequently, an Edge-Driven Feature Enhancement Network (ED-FEN) is integrated into the backbone, and a Local Deformable Attention (LDA) module is incorporated into the detection head. These additions are specifically engineered to bolster the model’s perception of small target edges and enhance its robustness to geometric distortions, culminating in the development of a high-precision model, NSE-YOLO. Finally, to achieve model lightweighting, a channel pruning strategy based on BatchNorm scaling factors is employed to compress the NSE-YOLO, yielding the ultimate LNSE-YOLO model. Experimental results on the TT100K and CCTSDB datasets demonstrate that, compared to the baseline YOLOv11n, our high-precision NSE-YOLO model achieves significant improvements in mAP@50 by 5.5 and 2.5 percentage points, respectively. The pruned LNSE-YOLO model, while maintaining its accuracy advantage, exhibits a substantial reduction in parameters and comparable computational cost to the baseline. This unequivocally validates the effectiveness and practical utility of our proposed methodology.

## 1 Introduction

The rapid development of Intelligent Transportation Systems (ITS) and autonomous driving technologies has made travel safer and more efficient. Among these advancements, the autonomous environmental perception capability of vehicles serves as the cornerstone for achieving high-level autonomous driving. Among various perception tasks, Traffic Sign Detection (TSD) plays an indispensable role. It is responsible for accurately and promptly recognizing critical traffic regulations, such as speed limits, prohibitions, and warnings, from complex visual inputs. The performance of TSD directly impacts driving safety, path planning, and decision-making reliability.

However, TSD faces numerous challenges in real-world traffic scenarios: (1) **Multi-scale Problem** [1]: Traffic signs exhibit significant size variations due to distance differences. Small distant targets, characterized by few pixels and weak features, are prone to missed detections; (2) **Complex Background Interference** [2]: Traffic signs often blend with billboards, buildings, trees, etc., demanding strong anti-interference capabilities from detection models; (3) **Geometric Deformation** [3]: Shooting angles, lens distortion, or physical damage to the signboards often lead to perspective, rotation, or non-rigid deformations, increasing detection difficulty; (4) **Deployment Constraints** [4]: Limited computational resources and power consumption of in-vehicle edge devices require detection models to possess both high efficiency and low complexity.

To address these issues, numerous scholars have achieved significant progress by leveraging deep learning methods from various perspectives. In particular, single-stage detectors represented by the YOLO (You Only Look Once) series have become mainstream techniques in the TSD field due to their outstanding balance between detection speed and accuracy. However, when deploying these models, which perform well on standard datasets, onto in-vehicle edge devices with stringent limitations on computational resources, memory, and power consumption, they still face the following challenges in coping with dynamic and complex real-world driving environments.

Firstly, small and scale-varying targets hinder detection accuracy. Traffic signs exhibit significant size variations depending on their distance from the vehicle. Distant signs may occupy only a few pixels in the image, and their weak features are easily lost after successive downsampling in standard convolutional networks. Li et al. [5] proposed a Multi-Scale Feature Fusion (MFF) method to improve dense small object detection accuracy by designing a Scale-Aware Feature Fusion Network (SAFFNet), effectively integrating multi-scale features to retain more information on small objects. Yin et al. [6], based on YOLOv8, designed an efficient multi-scale feature fusion structure in the neck network—Small Object Bi-directional Feature Pyramid Network (SO-BiFPN), which enhanced information exchange between different feature layers. Zhao et al. [7] replaced YOLOv5s’s feature extraction with a hybrid of CSPNet and Faster-Net, reducing feature redundancy while improving detection accuracy. Li et al. [8] designed an optimized feature fusion module—ACFPN, integrating it with Faster R-CNN to improve the detection accuracy of small traffic signs.

Secondly, the complexity and dynamics of the background introduce substantial perceptual interference. In real driving scenarios, traffic signs are often partially occluded by other vehicles, roadside trees, or buildings. More challengingly, billboards and shop signs in the environment share high visual similarity with traffic signs in color and shape, easily leading to confusion and false detections by models. In response, Ma et al. [9] proposed TSD-Net, introducing a Feature Enhancement Module (FEM), a high-resolution detection branch, and an Adaptive Dynamic Feature Fusion (ADFF) detection head to address complex background interference. He et al. [10] proposed NTS-YOLO, which incorporated a Convolutional Block Attention Module (CBAM) and an Optimal Transport Assignment (OTA) loss function to effectively handle complex background interference at night. CBAM enhances the perception of traffic sign shapes by optimizing channel and spatial feature weights, thereby improving recognition accuracy under low light, complex backgrounds, and uneven illumination conditions. Zhang et al. [11] designed TSD-DETR, addressing background interference through a multi-scale feature extraction module and an efficient multi-scale attention mechanism.

Lastly, geometric deformations caused by viewpoint changes and physical damage constitute another major obstacle. Non-frontal shooting angles result in perspective and affine transformations, while bending, damage, or tilting of signboards introduce non-rigid deformations. Standard convolutional kernels, due to their fixed geometric structure, struggle to effectively capture and adapt to these irregular shape variations, which are critical for improving detection robustness. Although prior research has addressed this issue through deformable convolution mechanisms, such methods incur substantial computational overhead, limiting their application in lightweight models. Zhong et al. [12] proposed SWP-DETR, introducing the PradatorConv (PdConv) module to adaptively learn horizontal and vertical deformations, addressing geometric deformation problems in images.

The above methods have achieved significant improvements in detection accuracy but also brought substantial increases in model parameters and computational load. Considering that models need to be deployed on edge devices with strict limitations on computational resources and storage space, this paper proposes a Noise Suppression and Semantic Enhancement YOLOv11n Traffic Sign Detection algorithm (LNSE-YOLO), aiming to build a detector that achieves a better balance between high precision and high efficiency.

For improving detection accuracy, we introduce innovations from three perspectives: (1) designing an NSSE module to enhance feature representation for small objects while suppressing noise; (2) introducing an Edge-Driven Feature Enhancement Network (ED-FEN) to strengthen the edge features of blurred or low-contrast targets; (3) integrating a Local Deformable Attention (LDA) module to better adapt to various geometric deformations of targets. In terms of improving deployment efficiency, we further apply a channel pruning strategy to systematically compress the model. Through these improvements, we construct a traffic sign detection model that achieves an optimal balance between detection accuracy and computational efficiency.

## 2 Related work

The YOLO (You Only Look Once) series of algorithms has been widely applied in the field of object detection due to its exceptional balance between speed and accuracy. As a representative of this series, YOLOv11 [13] has achieved significant performance improvements by introducing a new network structure and anchor-free design. However, its standard version remains too large for resource-constrained platforms. Although the lightweight version, YOLOv11n, achieves faster inference speed, its detection accuracy in complex scenarios, particularly for small objects and deformed targets, still requires enhancement [14,15].

In 2016, Redmon et al. [16] proposed the YOLO object detection framework, which divides the input image into a fixed number of grid cells and performs simultaneous category prediction and bounding box localization within each cell, achieving high detection efficiency. Nevertheless, this method exhibits limitations in detecting small objects, and there remains room for improvement in detection accuracy. In the same year, Liu et al. [17] released the Single Shot MultiBox Detector (SSD), which utilized multi-scale feature maps to adapt to objects of different sizes, significantly alleviating the problem of detecting small targets. Subsequently, YOLOv2 [18] and YOLOv3 [19] were introduced, incorporating anchor box mechanisms, feature fusion strategies, and batch normalization techniques to further enhance model accuracy and stability. Additionally, RefineDet improved detection performance to a certain extent by refining anchor box regression and correction.

In 2020, YOLOv4 [20] combined multiple optimization strategies to further improve detection accuracy while maintaining a high frame rate. To overcome the structural limitations of anchor-based methods, researchers have successively proposed anchor-free detection architectures, such as CornerNet [21], CenterNet [22], and FCOS [23]. These approaches typically regress the object’s center points or boundary information for localization, achieving excellent performance in specific application scenarios.

In recent years, the YOLO series has continued to iterate and evolve. YOLOv7 [24] and YOLOv8 have built upon the core advantages of their predecessors, introducing new network structures and module designs to further enhance detection performance and inference speed. They also support multi-task scenarios such as object segmentation and pose estimation. The subsequent YOLOv9 [25], YOLOv10 [26], YOLOv11 [13], and YOLOv12 [27] mainly focus on local optimizations within the YOLOv8 framework, aiming to reduce parameters and computational complexity while maintaining high detection performance.

## 3 Methodology

**Fig 1:**
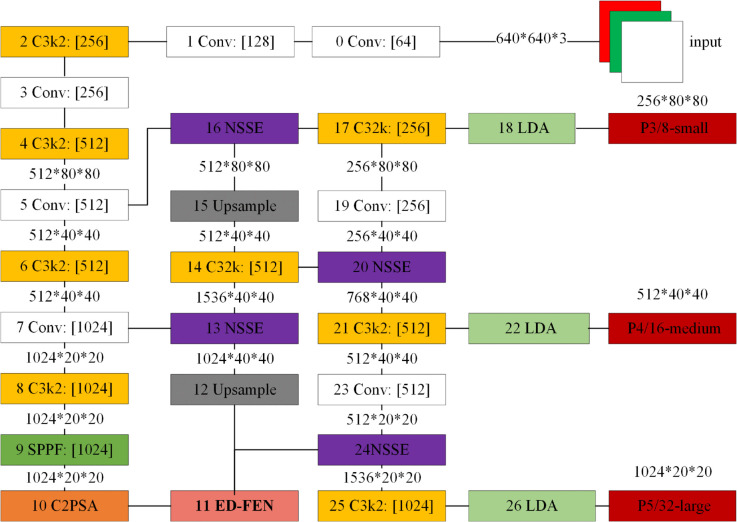
NSE-YOLO structure.

To enhance the detection accuracy of the YOLOv11 network in traffic sign detection tasks and effectively reduce model complexity, this paper proposes an improved model, NSE-YOLO, built upon the lightweight YOLOv11n version. The overall architecture of NSE-YOLO is depicted in Fig 1. This model incorporates optimizations primarily across three dimensions: feature fusion, attention mechanisms, and model compression. Specifically, we introduce an optimized approach for small object detection in traffic signs. For object recognition, a Noise Suppression and Deep Semantic Enhancement Multi-scale Feature Fusion Module (NSSE) is designed to improve the detection capability for small targets. Concurrently, an Edge-Driven Feature Enhancement Network (ED-FEN) is proposed, which combines edge detection with self-attention mechanisms to enhance the edge feature representation of targets, thereby increasing detection accuracy in complex backgrounds. To address geometric deformations of targets, this paper integrates a Local Deformable Attention (LDA) module, which utilizes learned sampling offsets and bilinear interpolation to align deformed targets. Furthermore, to enhance inference efficiency, a channel pruning method is employed to reduce redundant computations, ensuring both high efficiency and precision.

### 3.1 Multi-scale feature fusion with Noise Suppression and Deep Semantic Enhancement (NSSE)

**Fig 2:**
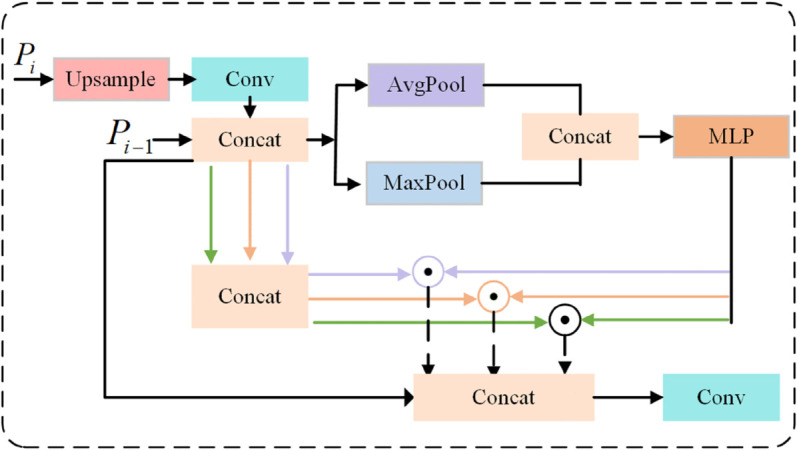
The NSSE module.

Accurate identification of traffic signs is a pivotal step in ensuring driving safety. However, traffic signs typically manifest as small objects in images and are susceptible to interference from complex backgrounds and varying lighting conditions, rendering traditional detection methods largely ineffective in addressing these challenges. To bolster the detection performance of small traffic sign targets, we propose a Multi-scale Feature Fusion Module combining Noise Suppression and Deep Semantic Enhancement (NSSE). As illustrated in Fig 2, this module effectively enhances small target detection capabilities through the synergistic fusion of deep and shallow features, the incorporation of multi-scale convolutions, and the design of a channel-adaptive attention mechanism. The design and implementation of this module are detailed below.

#### 3.1.1 Fusion and upsampling of deep and shallow features.

In Convolutional Neural Networks (CNNs), shallow feature maps typically capture rich local detail information, whereas deep feature maps encapsulate broader contextual information. In traffic sign detection images, a significant number of minute targets exist, whose features are progressively lost as they undergo downsampling through the backbone network.

To fully leverage both the detailed features from shallow layers and the contextual information from deep layers, thereby preserving more shallow-layer feature information, we perform an initial fusion of features from two adjacent layers in the backbone network. Specifically, a deep feature map *P*_*i*_ is upsampled to match the dimensions of a shallower feature map *P*_*i*−1_, following which *P*_*i*−1_ and *P*_*i*_ are concatenated to provide a rich feature input for the neck network.

$$P_{fused}=Concat(P_{i}^{upsampled},P_{i-1})$$ (1)

#### 3.1.2 Multi-scale feature extraction.

Building upon the merged feature map *P*_*fused*_, convolutional kernels of varying sizes are applied to extract features at different receptive fields. Specifically, convolutional kernels of size 3×3, 5×5, and 7×7 are employed in parallel to perform multi-scale feature extraction.

Let *H*_1_, *H*_2_, and *H*_3_ denote the output feature maps obtained by applying convolution operations with kernel sizes 3×3, 5×5, and 7×7 respectively on *P*_*fused*_:

$$H_{1}={\text{Conv}}_{3\times 3}(P_{fused})$$ (2)

$$H_{2}={\text{Conv}}_{5\times 5}(P_{fused})$$ (3)

$$H_{3}={\text{Conv}}_{7\times 7}(P_{fused})$$ (4)

Through these parallel convolution operations, features at multiple scales are extracted to capture multi-level information ranging from fine details to global context. This enables the model to adapt to traffic signs of different sizes and effectively extract target-relevant features.

#### 3.1.3 Adaptive noise suppression mechanism.

Inspired by the CBAM attention mechanism, this paper proposes an improved Adaptive NSSE module. Unlike CBAM, which sequentially combines channel and spatial attention, NSSE retains only the channel-wise adaptive weighting mechanism and introduces multi-scale convolutional feature fusion before the attention computation to strengthen semantic representations of small objects under different receptive fields. Moreover, the design goal of NSSE is not a general feature recalibration, but rather a targeted suppression of feature channels containing background noise or redundant information, enabling more discriminative feature refinement. In addition, unlike traditional attention modules, NSSE explicitly fuses adjacent-layer features, complementing shallow spatial information with deeper semantic cues to construct a more powerful multi-scale feature representation.

Firstly, Global Pooling and Average Pooling operations are applied to the multi-scale convolved feature maps to extract global contextual information from each channel. Assuming the feature map has *C* channels, the global information representations for each channel after pooling are obtained as follows:

$$F_{1}=MaxPooling(P_{fused})$$ (5)

$$F_{2}=AveragePooling(P_{fused})$$ (6)

Next, the pooled feature vectors are passed through a shared Multi-Layer Perceptron (MLP), which generates the channel attention weights *W* based on the pooled information:

$$W=\sigma \left(MLP\left(concat(F_{1},F_{2})\right)\right)$$ (7)

where σ denotes the Sigmoid activation function, and *W* represents the channel attention weights. Each channel’s weight *W*_*i*_ indicates its contribution to target detection.

Subsequently, each multi-scale convolved feature map $H_{1},H_{2},H_{3}$ is multiplied by its corresponding attention weight $W_{1},W_{2},W_{3}$, resulting in the weighted feature maps $M_{1},M_{2},M_{3}$:

$$M_{i}=W_{i}\cdot H_{i},\;i=1,2,3$$ (8)

#### 3.1.4 Feature concatenation and output.

Finally, the feature maps $M_{1},M_{2},M_{3}$, which have been adaptively weighted through channel attention, are concatenated to integrate information from different scales. This concatenation operation effectively fuses key features extracted at multiple scales, resulting in the final fused feature map *M*:

$$M=Concat(M_{1},M_{2},M_{3})$$ (9)

By combining deep-shallow feature fusion, multi-scale convolution, and the adaptive channel attention mechanism, the proposed method significantly enhances the accuracy of small traffic signal detection. The fusion of deep and shallow features ensures the effective combination of fine-grained details and contextual information. The multi-scale convolution enables the model to adapt to traffic signals of varying sizes, while the adaptive channel attention mechanism dynamically adjusts channel weights based on their contributions, thereby improving the representation capability of critical features.

### 3.2 Edge-Driven Feature Enhancement Network (ED-FEN)

In the context of traffic sign detection, a prevalent challenge is the blending of target objects with the image background, leading to insensitivity to target edge information. To effectively counter this, this paper proposes an Edge-Driven Feature Enhancement Network (ED-FEN). This network, through an edge-driven mechanism, automatically captures and strengthens edge information within the image, particularly under complex background and low-contrast conditions, thereby improving target detectability. Specifically, the ED-FEN integrates a designed edge enhancement technique with feature fusion strategies, enabling it to extract and reinforce target edge features at different hierarchical levels. Through feature concatenation, it augments the detailed information within the image, thereby enhancing the algorithm’s detection capability for small targets like traffic signs, especially when the boundaries between objects and backgrounds are indistinct or information is unclear.

#### 3.2.1 Edge Feature Enhancement Module (EFEM).

To overcome the limitations of traditional methods in complex backgrounds and to strengthen the representation of critical edge details, thereby improving the recognition rate and accuracy of small traffic signal targets, an Edge Feature Enhancement Module (EFEM) is designed to achieve precise edge information extraction. The specific process is as follows:

**Adaptive pooling.** Firstly, an adaptive pooling operation is applied to the input image to eliminate low-frequency background information while preserving local structural details. Adaptive pooling dynamically adjusts the pooling window and stride to ensure that the edge information of small objects, such as traffic signals, is retained:

$$X^{\prime }=AdaptivePool(X)$$ (10)

where $X^{\prime }$ denotes the pooled output size.

**Edge detection and enhancement.** Secondly, to accurately capture the edge information of traffic signals, the Sobel edge detection operator is utilized to extract edge features from the image. By computing the image gradients in both horizontal and vertical directions, the Sobel operator effectively extracts edge information. The detected edge features are further enhanced through convolution operations to improve their prominence. The Sobel edge detection formulas are defined as:

$$G_{x}=[\begin{array}{ccc} -1 & 0 & 1\\ -2 & 0 & 2\\ -1 & 0 & 1 \end{array}]*X^{\prime }$$ (11)

$$G_{y}=[\begin{array}{ccc} -1 & -2 & -1\\ 0 & 0 & 0\\ 1 & 2 & 1 \end{array}]*X^{\prime }$$ (12)

The edge map *G* obtained from the above computations highlights the edge features of traffic signals. The gradient magnitude is calculated as:

$$G=\sqrt{G_{x}^{2}+G_{y}^{2}}$$ (13)

**Self-attention mechanism.** Next, a Self-Attention Mechanism is introduced to enhance the features of important regions within the image. This mechanism learns the relationships among different regions of the image, enabling adaptive focus on the areas where traffic signals are located, thereby effectively enhancing the edge information in these regions. The self-attention mechanism is formulated as:

$$Attention(Q,K,V)=softmax\left(\frac{QK^{T}}{\sqrt{d}}\right)V$$ (14)

where *Q*, *K*, and *V* represent the query, key, and value matrices, respectively, and *d* is the feature dimension. By computing the similarity between regions, the attention mechanism dynamically assigns weights to emphasize the parts of the image that contain significant information. This allows the model to effectively focus on traffic signal areas and enhance their edge features, improving detection precision. In addition, to achieve a closer integration between edge detection and the attention mechanism, the edge map *G* obtained from the Sobel operator is introduced into the attention computation to guide the distribution of attention weights. Specifically, the edge map is normalized and processed through a 1 × 1 convolution to generate an edge weight map *E*, which is then incorporated into the attention calculation as follows:

$$Attention_{edge}(Q,K,V)=softmax\left(\frac{QK^{T}}{\sqrt{d}}⊙E_{ref}\right)V$$ (15)

where *E*_*ref*_ denotes the resized edge weight map aligned with the spatial dimensions of the attention matrix, and ⊙ represents element-wise multiplication. This Sobel-guided attention effectively enhances the feature responses around target boundaries while suppressing background noise, leading to clearer and more discriminative edge representations for small traffic signs. The visualization results in Fig X demonstrate that the proposed edge-guided attention significantly strengthens edge details compared to the baseline attention mechanism.

**Channel attention mechanism (SE Block).** Subsequently, a Channel Attention Mechanism (SE block) is incorporated to further enhance the module’s focus on critical feature channels. By adaptively learning which channels are most relevant for traffic signal detection, the model improves its perception of key edge features while suppressing irrelevant information. The operation is defined as:

$$Enhanced\_edge=X\cdot \sigma (f_{SE}(X))$$ (16)

where *f*_*SE*_(*X*) represents the channel attention weights computed via fully connected layers and activation functions, and σ denotes the Sigmoid activation function.

**Feature fusion.** Finally, the enhanced edge features are fused with the original image through an addition operation to ensure the effective integration of both structural and edge-enhanced information. The fusion is formulated as:

$$X_{edge}=input+Enhanced\_edge$$ (17)

where *input* is the original image and Enhanced_edge is the edge-enhanced feature map. This addition operation ensures that the final output retains the original structure while emphasizing edge details.

#### 3.2.2 Edge-Driven Feature Enhancement Network (ED-FEN) Design.

**Fig 3:**
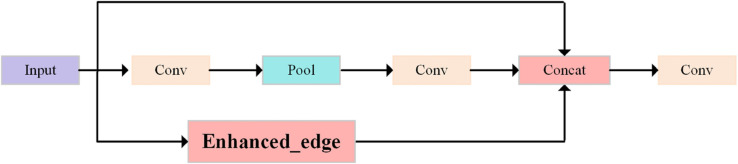
Edge-driven feature enhancement network.

In traffic signal detection tasks, complex backgrounds, low-contrast conditions, and the blending of target objects with their surroundings make it challenging to extract clear edge information. To address these issues, an Edge-Driven Feature Enhancement Network (ED-FEN) is proposed, as illustrated in Fig 3.

The ED-FEN is designed to enhance edge information and fine-grained features within images, aiming to significantly improve the representation of image details. This is particularly beneficial in scenarios where the target edges are not prominent, thereby increasing the model’s sensitivity to small targets.

By strengthening the extraction and enhancement of edge-related information, the ED-FEN effectively mitigates the adverse impact of background interference and blurry object boundaries, enabling more accurate detection of small traffic signals embedded in complex scenes.

The proposed Edge-Driven Feature Enhancement Network (ED-FEN) consists of the following stages:

**Preprocessing of input features.** Initially, the input image features undergo a preliminary convolution operation to reduce dimensionality and enhance feature representation capability:

$$X^{\prime }=Conv(X)$$ (18)

where *X* denotes the input feature map, and $X^{\prime }$ is the processed feature map.

**Multi-scale processing and pooling operation.** At the intermediate stage, average pooling is applied to the feature map to downsample its spatial dimensions, enabling multi-scale feature extraction. Pooling effectively preserves key information in the image while suppressing background noise:

$$X_{pool}=AvgPool(X^{\prime },\;kernel=3,\;stride=1,\;padding=1)$$ (19)

**Edge Feature Enhancement Module (EFEM).** To improve the accuracy of traffic signal detection while maintaining computational efficiency, an Edge Feature Enhancement Module (EFEM) is introduced. This module utilizes an attention mechanism to amplify features in important regions, significantly enhancing the expression of edge information for traffic signals. Detailed implementation of EFEM is described in Sect 3.2.1.

**Feature concatenation and fusion optimization.** After adaptive pooling and edge feature enhancement, the extracted key edge features and enhanced features are concatenated and fused with the original input features. The feature concatenation operation is defined as:

$$X_{fuse}=Concat(X_{pool},X_{edge},X)$$ (20)

where *X*_*pool*_ is the pooled feature map, *X*_*edge*_ is the edge-enhanced feature map obtained from EFEM, and *X* is the original input feature map.

**Final feature mapping.** Finally, a 1×1 convolution layer is employed to map the fused feature map back to the original number of input channels, ensuring efficient compression and integration of information:

$$X_{final}=Conv(X_{fuse})$$ (21)

### 3.3 Local Deformable Attention (LDA)

To better accommodate the geometric deformation of traffic signs in complex traffic scenarios, such as variations in camera angle and lens distortion, this paper proposes a Local Deformable Attention (LDA) module. This module aims to enhance the model’s perception of local geometric changes in targets and is particularly well-suited for detecting deformed small objects. The LDA module primarily comprises three components: deformable offset estimation, feature sampling alignment, and weighted fusion enhancement.

Given an input feature map $F\in {\mathbb{R}}^{C\times H\times W}$, a 3 × 3 convolution layer is first used to predict the sampling offsets for each spatial position:

$$\Delta p=Conv_{3\times 3}(F)$$ (22)

where $\Delta p\in {\mathbb{R}}^{2N\times H\times W}$ represents the 2D coordinate offsets for *N* sampling points.

For each standard sampling location *p*_*i*_, the actual sampling location is computed as:

$${\hat{p}}_{i}=p_{i}+\Delta p_{i}$$ (23)

Considering that ${\hat{p}}_{i}$ is generally a non-integer coordinate, bilinear interpolation is adopted to extract the continuous feature value from the original feature map *F* at the deformed sampling location:

$$F^{D}(p_{i})=\sum_{q\in 𝒩({\hat{p}}_{i})}w(q,{\hat{p}}_{i})\cdot F(q)$$ (24)

where $𝒩({\hat{p}}_{i})$ denotes the set of four neighboring integer grid points surrounding ${\hat{p}}_{i}$, and w(·) represents the bilinear interpolation weights.

To enhance the module’s capability of representing local deformations, a channel attention mechanism is applied to the deformation-aligned feature map *F*^*D*^. The attention weights *A* are computed as:

$$A=Softmax(MLP(AvgPool(F^{D})))$$ (25)

The final enhanced feature map is obtained by performing channel-wise weighted multiplication:

$$F^{\prime }=A\cdot F^{D}$$ (26)

where $A\in {\mathbb{R}}^{C\times 1\times 1}$ denotes the channel attention weights, and · indicates channel-wise multiplication.

The LDA module introduces learnable sampling offsets and a localized attention mechanism, effectively addressing the limitations of conventional fixed receptive field methods in handling geometric variations of targets. This design is particularly beneficial for small object detection tasks such as traffic signal lights, enabling the detection head to adaptively enhance its response to small and deformed objects in complex traffic scenarios. By precisely modeling local geometric deformations, the LDA module significantly improves the overall detection performance, especially in dynamically changing traffic environments, where it effectively mitigates issues caused by spatial and image distortions of traffic signal lights.

The proposed Local Deformable Attention (LDA) module is conceptually related to Deformable Convolutional Networks (DCN) and deformable attention mechanisms such as Deformable-DETR, yet it differs significantly in both design objectives and computational granularity: (1) Sampling scope: DCN predicts offsets for each convolution position, while Deformable-DETR predicts sparse sampling points for each query across multi-scale features. In contrast, LDA constrains offset learning within a local neighborhood (e.g., a 3 × 3 or 5 × 5 window), achieving fine-grained geometric adaptation at lower computational cost. (2) Computational efficiency:Global deformable attention requires multi-head interpolation across scales, leading to high computational overhead. LDA performs deformable sampling only within local regions and shares offset parameters across heads, thereby reducing computational complexity while maintaining or even improving accuracy. (3) Attention coupling:LDA applies channel attention immediately after deformable sampling to strengthen critical features, whereas existing methods typically decouple the deformable operation and attention computation. Overall, this design enables LDA to capture local geometric deformations with lightweight computation, making it particularly suitable for real-time small object detection scenarios.

### 3.4 Channel pruning

To further enhance the inference efficiency of the model on edge devices, this paper, after completing structural optimization, employs a channel pruning method to compress the proposed NSE-YOLO model, thereby reducing redundant computations and increasing operational speed. This method achieves simultaneous compression of parameters and computational load by evaluating channel importance and selectively removing channels that have minimal impact on model performance.

We utilize the scaling factor γ in Batch Normalization (BN) layers as an indicator of channel importance. Given a feature map $F\in {\mathbb{R}}^{C\times H\times W}$, its output after passing through a BN layer is expressed as:

$$F_{BN}=\gamma \cdot \frac{F-\mu }{\sqrt{\sigma^{2}+\epsilon }}+\beta$$ (27)

where γ is the learnable scaling factor for each channel, μ and $\sigma^{2}$ are the mean and variance, respectively, ϵ is a small constant for numerical stability, and β is the shift parameter.

The absolute values of γ are sorted in ascending order, and a pruning ratio α is set. The channels corresponding to the smallest αC scaling factors are selected as pruning candidates. Subsequently, the low-importance channels are pruned, resulting in a compressed feature map:

$$F^{\prime }=Prune(F,\gamma ,\alpha )$$ (28)

where Prune(·) denotes the operation of retaining the top 1−α proportion of important channels. After pruning, fine-tuning is necessary to restore the model’s performance on the compressed structure.

To mitigate the abrupt performance drop caused by aggressive one-shot pruning, a staged pruning strategy is adopted. After each pruning round, a short cycle of fine-tuning is performed, gradually converging the pruned model to a stable state. Let the total number of pruning stages be *n*, with each stage having a pruning ratio of $\alpha_{i}$, the overall model compression ratio (CR) after all stages is given by:

$$CR=1-\prod_{i=1}^{n}(1-\alpha_{i})$$ (29)

where *CR* denotes the cumulative compression ratio.

As an essential model compression technique, channel pruning significantly reduces the number of parameters and computational cost of the NSE-YOLO model. Coupled with the structural optimizations proposed earlier, the pruned model maintains high detection accuracy while greatly enhancing deployment efficiency and real-time performance on edge devices.

## 4 Experiments and results analysis

### 4.1 Experimental datasets

This study utilizes the TT100K dataset [28] for comparative experiments and validates generalization performance on the CCTSDB2021 dataset [29].

**Fig 4:**
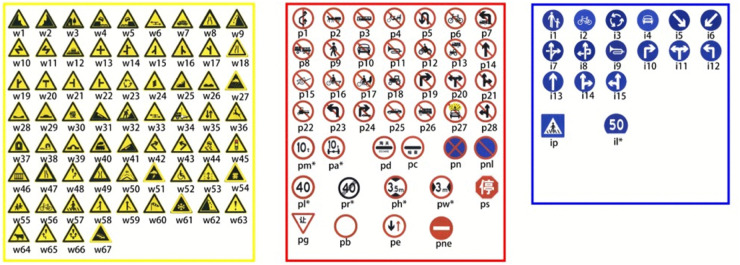
Categories included in the TT100K dataset.

TT100K, released by a Chinese traffic research institution, is a large-scale traffic sign detection dataset designed for recognition and detection tasks. It contains over 100,000 images captured under diverse traffic scenarios, making it suitable for training and testing deep learning models. The dataset features complex conditions, including variations in lighting, weather, and road environments, detailed annotations for more than 15 traffic sign types such as speed limit, prohibitory, and warning signs, and coverage of urban roads, highways, and rural areas. Originally, TT100K includes three main categories—indicative, prohibitory, and warning—comprising 221 fine-grained subcategories. To mitigate class imbalance, a Python script was used to filter 42 categories with at least 100 samples each. The filtered dataset was then split into training and test sets at a ratio of 7:2, resulting in 7,773 training images and 1,965 test images. The original dataset encompasses three major traffic sign categories—indicative, prohibitory, and warning—totaling 221 fine-grained subcategories, as depicted in Fig 4. To address the issue of unbalanced data distribution, this study filtered the data using a Python script, ultimately selecting 42 traffic sign categories, each with over 100 samples, as the subjects of research. After filtering, the dataset was partitioned as follows: the training set contains 7,773 images, and the test set contains 1,965 images.

The CCTSDB2021 dataset contains 17,856 images, with traffic signs divided into mandatory, prohibitory, and warning classes. It encompasses diverse weather conditions, lighting variations, road backgrounds, and nearly 40 traffic sign types commonly found in China, ensuring high diversity and practical relevance. Following the original dataset protocol, it was divided into a training set of 16,356 images and a test set of 1,500 images,In snowy conditions, signs may appear whitish due to overexposure; in foggy conditions, water mist causes hazy scenes and blurred signs; in rainy conditions, dark environments and reflections blur both windshields and signs. Image counts are: sunny 400, cloudy 300, nighttime 500, snow 100, fog 40, and rain 160. This distribution enables robust evaluation under varying illumination and extreme conditions. The datasets were split proportionally to maintain a balanced representation of each category, with an approximate 7:2:1 ratio for training, validation, and test sets, ensuring reproducibility of the experiments.

### 4.2 Experimental environment and parameters

The experiments in this paper are developed using the PyTorch deep learning framework. The experimental environment is shown in Table 1.

**Table 1:** Experimental environment setting.

Experimental Environment	Configuration
CPU	Xeon E5-2667 V3
GPU	NVIDIA RTX 3090 24G
Memory	32G
Programming Language	Python 3.9
Deep Learning Framework	PyTorch 2.6.0 + cu118

For hyperparameter settings during model training, this study selects the Adam optimizer for parameter updates, with a base learning rate configured at 0.0001. The batch size is set to 8 during training, and the model is optimized over 300 epochs. All input images are preprocessed to a uniform size of 640×640 pixels. To ensure the reliability of experimental comparisons, different experimental groups adopt exactly the same set of hyperparameters, thereby eliminating the impact of training condition differences on result analysis.

For data augmentation, this study utilizes the default strategies provided by the YOLOv11 framework to improve model generalization and detection performance. Specifically, Mosaic augmentation randomly combines four images into one to enhance the detection of small and dense targets; MixUp is applied with a low ratio to generate blended samples, improving robustness; HSV color jittering perturbs hue, saturation, and value to increase resilience to lighting variations; and random affine transformations, including scaling, translation, and rotation, enhance the model’s adaptability to different viewpoints and geometric deformations.

### 4.3 Evaluation metrics

This study adopts a multi-dimensional evaluation system to quantitatively analyze the detection performance of the model, focusing on the following core metrics: Precision, Recall, mean Average Precision (mAP), number of model parameters (Params), and floating point operations (FLOPs). The definitions and calculation methods of each metric are as follows:

Precision is an important metric to evaluate the accuracy of model predictions, defined as the proportion of true positive samples among all samples predicted as positive. The mathematical expression is:

$$\text{Precision}=\frac{TP}{TP+FP}$$ (30)

where *TP* (True Positives) is the number of correctly detected positive samples, and *FP* (False Positives) is the number of negative samples mistakenly detected as positive.

Recall is a key metric to evaluate the model’s ability to identify positive samples, expressed as:

$$\text{Recall}=\frac{TP}{TP+FN}$$ (31)

where *FN* (False Negatives) is the number of positive samples that are missed by the model.

Mean Average Precision (mAP) is a core evaluation metric in multi-class object detection, defined as:

$$\text{mAP}=\frac{1}{N}\sum_{i=1}^{N}{\text{AP}}_{i}$$ (32)

where *N* is the total number of categories to be detected, and ${\text{AP}}_{i}$ represents the average precision of the *i*-th category.

The number of model parameters is an important indicator to assess the complexity of deep learning models, defined as:

$$\text{Params}=\sum_{l=1}^{L}(W_{l}+b_{l})$$ (33)

where *L* is the total number of layers in the model, *W*_*l*_ is the weight parameter matrix of the *l*-th layer, and *b*_*l*_ is the bias parameter vector of the *l*-th layer.

FLOPs (Floating Point Operations) is a key metric to measure the computational complexity of the model, representing the total number of floating point operations required for a single forward inference. This metric comprehensively reflects the computational resource requirements of the model, including basic operations such as multiplications and additions in convolutional layers, fully connected layers, etc.

### 4.4 Comparative experimental results

**Fig 5:**
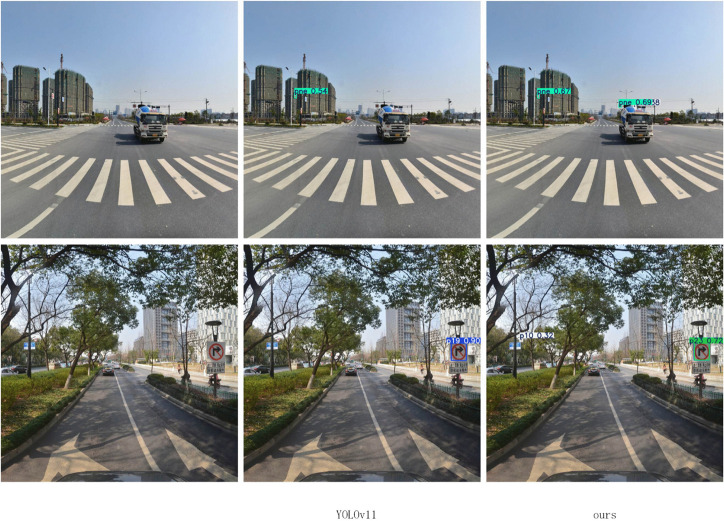
Improvement on missed detections of occluded targets.

**Fig 6:**
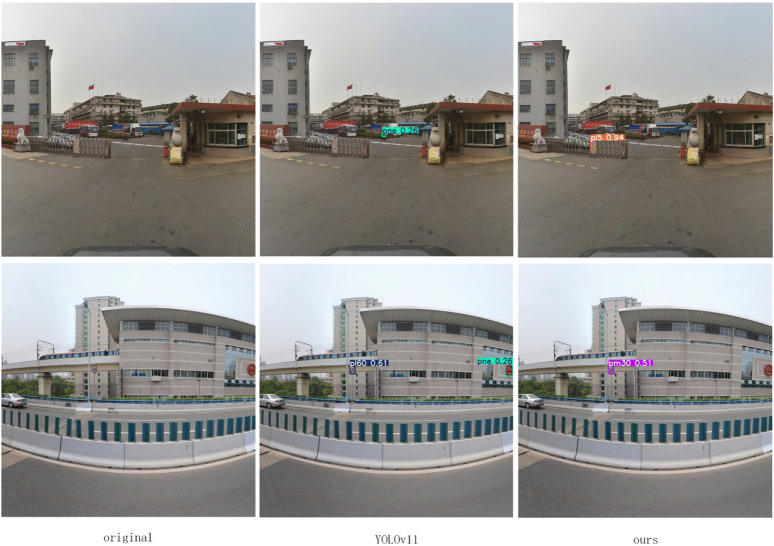
Improvement on misdetections.

**Fig 7:**
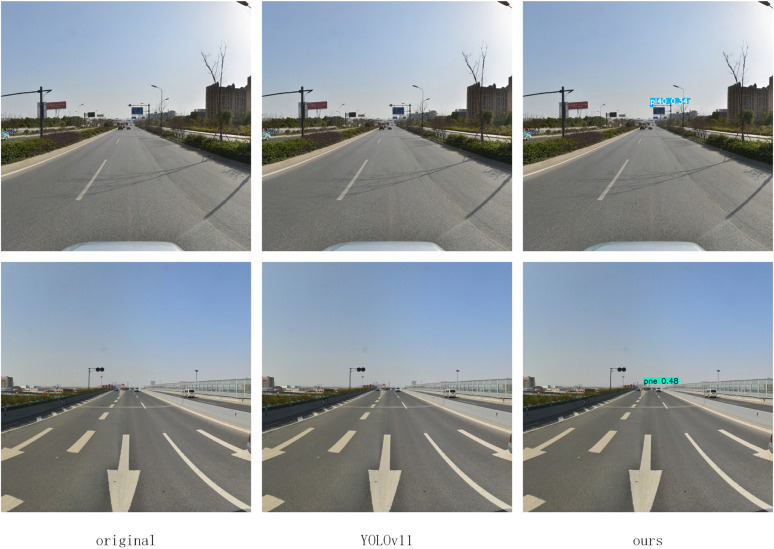
Improved misdetection of small targets at long range.

**Fig 8:**
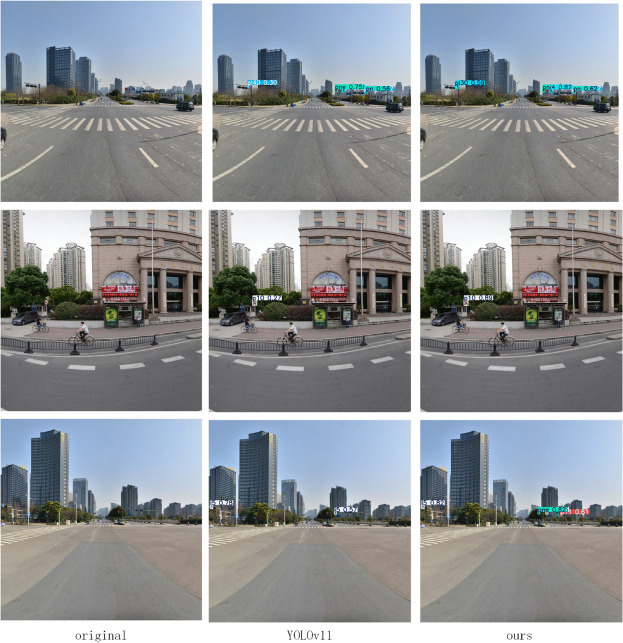
Improvement on missed detections of small targets at long range.

To comprehensively evaluate the performance of the proposed algorithm, this paper conducted a comparative analysis on the TT100K dataset against current mainstream lightweight object detection algorithms. The experimental results, presented in Table 2, indicate that our proposed LNSE-YOLO model demonstrates significant advantages in both performance and efficiency. Compared to the high-precision NSE-YOLO, LNSE-YOLO exhibits a substantial reduction in both parameters and computational load, approaching the level of YOLOv9t. Furthermore, compared to the baseline YOLOv11n, LNSE-YOLO significantly surpasses it in the mAP@50 metric, showcasing superior detection accuracy while having a comparable computational cost. In comparison with other mainstream lightweight object detection models (e.g., YOLOv9t and YOLOv10n), LNSE-YOLO consistently achieves the best mAP@50 score, highlighting its outstanding detection performance. To strengthen the comparison, FM-YOLOv11 and GFL were additionally included. FM-YOLOv11 integrates feature fusion and attention mechanisms and achieves high accuracy, but its computation (7.8M parameters, 26.3G FLOPs) remains far heavier than the proposed LNSE-YOLO (2.05M, 6.3G). GFL, a representative anchor-free detector, also shows strong performance but has an even larger model size (32.3M parameters, 128.8G FLOPs). These results further highlight the computational efficiency of the proposed approach. The effective channel pruning strategy employed in LNSE-YOLO significantly reduces computational overhead, further enhancing its deployment feasibility in resource-constrained scenarios. Visual improvements in detecting occluded, misclassified, and distant small targets are illustrated in Figs 5–8.

**Table 2:** Comparative experimental results on the TT100K dataset.

Model	Params/M	FLOPs/G	Precision/%	Recall/%	mAP@50-95/%	mAP@75/%	mAP@50/%
YOLOv8n	3.01	8.2	73.0	68.4	56.4	65.8	74.3
YOLOv9t	1.98	7.6	79.2	65.6	57.4	67.2	75.8
YOLOv10n	2.28	6.6	74.0	64.9	55.8	65.5	73.2
YOLOv11n	2.58	6.3	71.1	66.6	54.9	64.6	71.6
YOLOv12n	2.57	6.3	71.5	63.0	53.8	62.7	70.4
FM-YOLOv11 [30]	7.8	26.3	87.4	84	89.6	–	89.6
GFL [31]	32.3	128.8	–	–	48.0	–	64.6
NSE-YOLO	2.87	10.2	80.8	69.2	58.9	70.5	77.1
LNSE-YOLO	2.05	6.3	77.5	68.1	57.2	68.5	75.2

### 4.5 Pruning rate comparative experimental results

To systematically evaluate the impact of channel pruning on the NSE-YOLO model’s performance and computational efficiency, this paper conducted progressive pruning experiments on the TT100K dataset, using the unpruned NSE-YOLO (with an acceleration ratio of 1.0) as the baseline. The results are detailed in Table 3. The baseline NSE-YOLO has 2.87M parameters, 10.2G FLOPs, an mAP@50 of 77.1%, and an mAP@50-95 of 58.9%.

**Table 3:** Model pruning comparison experiments on the TT100K dataset.

Accel. Ratio	Params/M	FLOPs/G	Precision/%	Recall/%	mAP@50-95/%	mAP@75/%	mAP@50/%
1.0 (None)	2.87	10.2	80.8	69.2	58.9	70.5	77.1
1.6	2.16	7.53	78.3	69.5	57.8	70.1	74.3
1.8	2.05	6.3	77.5	68.1	57.2	68.5	75.2
2.0	1.92	5.95	78.6	68.4	57.4	69.5	73.9
2.2	1.83	5.62	77.3	67.2	55.8	65.5	72.6
2.4	1.78	5.46	76.1	66.6	54.9	64.6	71.3

The experimental results indicate that as the pruning acceleration ratio increases, both the model’s parameter count and computational load exhibit a continuous downward trend. When the acceleration ratio reaches 1.6, the model achieves significant lightweighting, with parameters and computational load reduced by 24.7% and 13.4% respectively, and an mAP@50 of 76.3%. Further increasing the acceleration ratio to 1.8 results in a 28.6% reduction in parameters and a substantial 27.6% reduction in computational load. At this configuration, the mAP@50 is 75.2%. While individual accuracy metrics show a slight decrease, the significant compression in computational load leads to an optimal balance between performance and efficiency. This configuration aligns with the parameters and FLOPs of the final LNSE-YOLO model (as shown in Table 2), thus confirming it as the chosen lightweighting solution.

**Fig 9:**
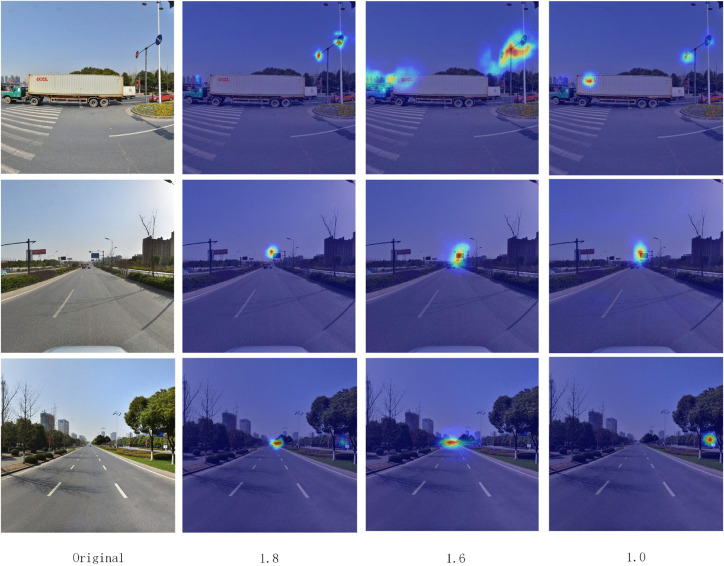
Feature map visualization of key network layers Under different pruning ratios.

When the acceleration ratio continues to increase to 2.0 and beyond, the model’s detection accuracy begins to decline notably. For instance, at an acceleration ratio of 2.4, the mAP@50-95 sharply drops to 54.9% and mAP@75 plunges by 5.9%, explicitly indicating that excessive pruning severely impairs the model’s feature representation capability and detection accuracy. To further verify how different pruning ratios affect the model’s ability to preserve key features, this study visualizes the feature maps of critical network layers under pruning ratios of 1.0, 1.6, and 1.8, using heatmaps to display activation intensity. As illustrated in Fig 9, the results show that the unpruned 1.0 model retains the most complete and informative feature representations, with concentrated and clearly structured responses. The 1.6-pruned model exhibits weakened activations in some high-level layers, with noticeable degradation in edge structures and texture details. Notably, the 1.8-pruned model still maintains activation patterns highly consistent with the 1.0 model, and its key object contours and feature response regions remain clearly identifiable. This indicates that even with substantial reductions in parameters and FLOPs, the model preserves strong feature representation capability, which aligns with the quantitative results showing only a slight performance drop compared to the 1.0 model. These visualization findings demonstrate that the proposed pruning strategy achieves an effective balance between lightweight design and accuracy retention.

In summary, the pruning experiments validate the effectiveness of channel pruning in reducing model complexity, demonstrating that an acceleration ratio of 1.8 achieves the maximum computational efficiency improvement while maintaining high accuracy.

### 4.6 Ablation study results

To quantitatively assess the individual and combined contributions of the proposed improved modules to the model’s overall performance, an ablation study was conducted on the TT100K dataset. The detailed results are presented in Table 4. The baseline model for this study is YOLOv11n, which exhibits an mAP@50 of 71.6%, a parameter count of 2.58M, and a computational load of 6.3GFLOPs.

**Table 4:** Detection results with different improvement strategies.

Experiment	ED-FEN	LDA	NSSE	mAP@50/%	Params/M	FLOPs/G
YOLOv11n (Baseline)				71.6	2.58	6.3
A	✓			74.1	3.23	7.9
B		✓		72.7	3.17	7.3
C			✓	75.2	2.72	8.5
D	✓		✓	76.4	2.85	9.6
E		✓	✓	76.3	2.76	9.3
F (NSE-YOLO)	✓	✓	✓	77.1	2.87	10.2

Note: ✓ indicates the module was used.

Firstly, the introduction of the Edge-Driven Feature Enhancement Network (ED-FEN) in Experiment A resulted in an mAP@50 improvement of 2.5 percentage points, increasing from the baseline’s 71.6% to 74.1%. This empirically demonstrates that ED-FEN, by strengthening edge information, effectively enhances the model’s detectability of traffic signs in complex backgrounds.

Secondly, the integration of the Local Deformable Attention (LDA) module in Experiment B led to an mAP@50 increase of 1.1 percentage points, reaching 72.7%. This indicates that LDA effectively boosts the model’s robustness to geometric deformations, with a relatively controllable increase in computational overhead.

Thirdly, the Noise Suppression and Semantic Enhancement (NSSE) module in Experiment C yielded the most significant single-module improvement, with mAP@50 reaching 75.2%, a 3.6 percentage point increase over the baseline. NSSE, by optimizing multi-scale feature fusion and suppressing background noise, substantially strengthens the feature representation of small targets.

Finally, when all three proposed modules (ED-FEN, LDA, and NSSE) were integrated to construct the high-precision NSE-YOLO model (Experiment F), its mAP@50 achieved 77.1%, representing a substantial 5.5 percentage point improvement over the baseline YOLOv11n. Although NSE-YOLO’s parameter count (2.87M) and computational load (10.2GFLOPs) show an increase compared to the baseline, the synergistic effects among the modules significantly amplify the detection accuracy gains.

**Fig 10:**
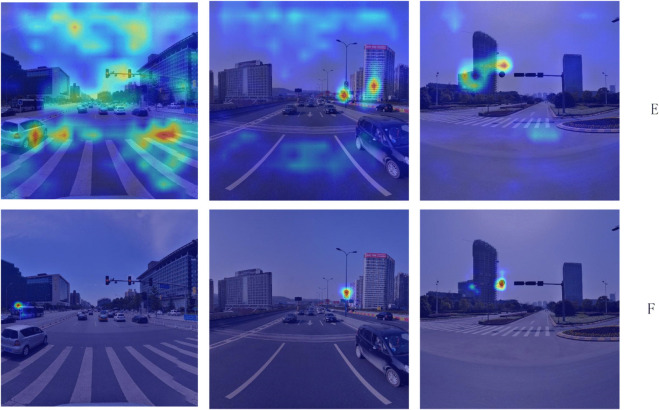
Visualization comparison of feature activation maps with and without the proposed ED-FEN module.

To further verify the effectiveness of the proposed ED-FEN module, a visual comparison of feature activation maps was conducted between Experiment E (without the ED-FEN module) and Experiment F (our improved model), as shown in Fig 10. The results show that the attention responses of Experiment E are scattered and significantly affected by background interference. In contrast, after integrating the ED-FEN module, the activation regions of the model become more concentrated on traffic signs and their boundary areas, while background responses are notably suppressed. This demonstrates that the Sobel-guided attention mechanism effectively enhances edge perception, thereby improving the detection performance for small and low-contrast targets.

This comprehensive ablation study unequivocally confirms the individual efficacy of each proposed module and their collective contribution to the overall performance enhancement, laying a robust foundation for subsequent model lightweighting through channel pruning.

### 4.7 Visualization of model decisions

**Fig 11:**
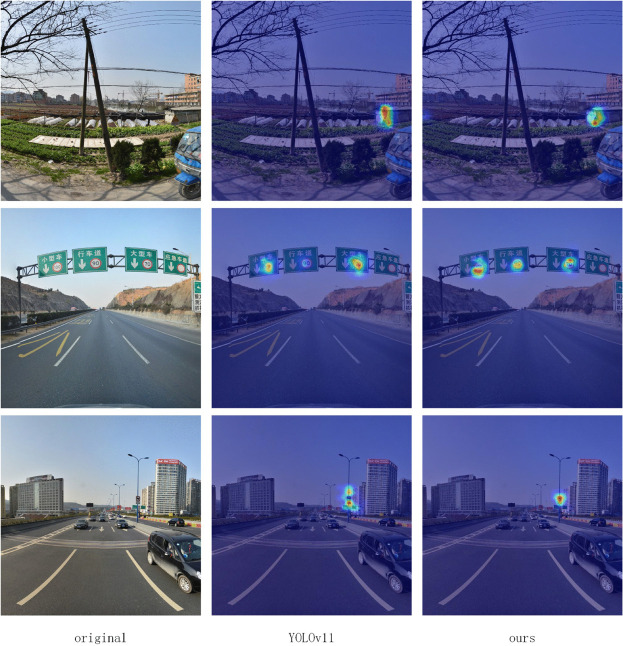
LayerCAM visualization.

To gain deeper insights into the model’s detection mechanisms, this study employed the LayerCAM visualization technique for interpretability analysis of the model’s decision-making process. As illustrated in Fig 11, this method generates heatmaps that intuitively represent the critical regions of focus for the model.

Observing the comparative visualization heatmaps of the two methods in Fig 11, it is evident that LNSE-YOLO exhibits superior detection performance compared to YOLOv11n. Particularly in scenarios involving distant targets and occlusions, LNSE-YOLO maintains a clear and concentrated heatmap response, whereas YOLOv11n demonstrates scattered or missing responses. These results validate that LNSE-YOLO, through improvements such as multi-scale feature fusion and attention mechanisms, significantly enhances the model’s perceptual capability for challenging samples, enabling it to sustain precise detection performance even in complex scenes. This qualitative visualization analysis further corroborates the effectiveness of the LNSE-YOLO architecture’s enhancements, complementing the quantitative experimental findings.

### 4.8 Generalization validation

To assess the adaptability of the LNSE-YOLO model across different datasets, we conducted generalization capability validation on the CCTSDB dataset. As shown in Table 5, on the CCTSDB dataset, the baseline YOLOv11n model achieved an mAP@50 of 79.1%. Our improved high-precision NSE-YOLO model (integrating all attention mechanisms and the NSSE module) reached an mAP@50 of 81.6%, demonstrating an effective improvement in detection accuracy, albeit with an increase in computational load. The final lightweight LNSE-YOLO model, while maintaining a robust mAP@50 of 80.3%, successfully reduced its parameter count to 2.05M and its computational load to 6.3 GFLOPs. More importantly, it achieved a real-time inference speed of **148 FPS** on the edge device, representing a significant improvement over the baseline YOLOv11n (131 FPS) and other lightweight models such as YOLOv10n (111 FPS) and YOLOv9t (122 FPS). This high processing speed confirms the suitability of LNSE-YOLO for real-time deployment on resource-constrained platforms. Overall, LNSE-YOLO achieves an excellent trade-off between accuracy and efficiency, exhibiting superior lightweight performance while preserving high detection precision. The comparative results further demonstrate the robustness and generalization capability of the proposed method across diverse datasets.

**Table 5:** Generalization experiment results on the CCTSDB2021 dataset.

Model	Params/M	FLOPs/G	Precision/%	Recall/%	mAP@50-95/%	mAP@75/%	mAP@50/%	FPS
YOLOv8n	3.01	8.2	89.2	69.3	50.9	59.2	79.8	158
YOLOv9t	1.98	7.6	86.6	71.8	48.9	55.2	77.5	122
YOLOv10n	2.28	6.6	85.4	70.3	51.1	59.9	78.4	111
YOLOv11n	2.58	6.3	90.1	69.6	50.9	58.9	79.1	131
YOLOv12n	2.57	6.3	88.3	70.4	51.6	61.0	78.8	73
NSE-YOLO	2.87	10.2	90.3	73.0	52.4	62.1	81.6	114
LNSE-YOLO	2.05	6.3	90.5	73.1	52.6	61.8	80.3	148

## 5 Conclusion

This paper addresses the challenges of insufficient accuracy and high computational overhead faced by existing traffic sign detection algorithms during deployment on edge devices by proposing LNSE-YOLO, a lightweight detection algorithm. Grounded in YOLOv11n as its baseline, the algorithm significantly enhances its detection capabilities for small, blurry, and geometrically deformed targets through the integration of a Noise Suppression and Semantic Enhancement module (NSSE), an Edge-Driven Feature Enhancement Network (ED-FEN), and a Local Deformable Attention module (LDA). Subsequently, a channel pruning technique is applied to compress the high-precision model, substantially reducing its complexity while preserving performance.

Experimental results on both the TT100K and CCTSDB datasets demonstrate that, compared to the unpruned NSE-YOLO, the pruned LNSE-YOLO exhibits a distinct advantage in both parameter count and computational efficiency. Furthermore, when compared to the baseline YOLOv11n, LNSE-YOLO achieves a significant reduction in parameters while maintaining a comparable computational load, thereby realizing a superior balance between detection performance and efficiency. In future work, further optimizations can be explored through more advanced compression techniques such as model quantization and knowledge distillation, as well as by enhancing the algorithm’s robustness to extreme weather conditions, to augment its practical application potential.
